# Software for dose adjustment of antimicrobials. Implications for plasma concentrations and pratical limitations

**DOI:** 10.31744/einstein_journal/2020CE5858

**Published:** 2020-10-20

**Authors:** Geisa Cristina da Silva Alves, Samuel Dutra da Silva, Farah Maria Drumond Chequer, Whocely Victor de Castro, André Oliveira Baldoni, Andras Farkas, Gergely Daróczi, Jason Alexander Roberts, Cristina Sanches

**Affiliations:** 1 Universidade Federal de São João del-ReiDivinópolisMGBrazil Universidade Federal de São João del-Rei , Divinópolis , MG , Brazil .; 2 Universidade de ItaúnaItaúnaMGBrazil Universidade de Itaúna , Itaúna , MG , Brazil .; 3 Optimum Dosing StrategiesBloomingdaleNJUnited States Optimum Dosing Strategies , Bloomingdale , NJ , United States .; 4 University of QueenslandQueenslandQLDAustralia University of Queensland , Queensland , QLD , Australia .

Dear Editor,

This letter is designed to complement data published by Silva et al., ^( 1 )^ presenting the results of plasma concentrations of patients who used piperacillin (PIP) in an empirical dose (ED) or dose adjusted (DA) by means of the software Optimum Dosing Strategies (ID-ODS). In addition, it describes some limiting aspects observed by the authors when using the software in the reality of a Brazilian hospital.

Currently, there are some types of software available worldwide for dose adjustment of antibiotics, which use specific data of the patient, together with pharmacokinetic population models. ^( 2 , 3 )^ Therefore, in case of no individual data on plasma concentrations available, the software can estimate individualized doses with high capacity and accuracy to achieve the target concentrations. ^( 2 , 3 )^ Moreover, it can be easily integrated to the medical record to enable clinical management of the critical patient, at bedside. ^( 2 , 4 )^ Hence, Silva et al., ^( 1 )^ translated and performed the linguistic and cultural validation of a dose adjustment software, with further randomized clinical trial comparing the outcome between the Groups ED and DA for PIP. The software was configured for the pharmacokinetic/pharmacodynamic (PK/PD) target, with 50% of time when the free concentrations were above the minimum inhibitory concentration (50%fT>MIC) and a MIC of 8mg/L.

The authors did not observe differences in the clinical outcomes between the groups. During the study, three blood samples were collected at day 5 of PIP treatment, at the same dose intervals, and they were later processed and analyzed. To evaluate microbiological efficacy, %fT>MIC was calculated considering plasma protein binding of 30% and extrapolating the decay curve for plasma MIC up to 8mg/L, based on the formula K _el_ = (lnC _1_ -lnC _2_ )/(T _2_ -T _1_ ), in which Kel is the elimination rate constant, and C1 and C2 are the plasma concentrations at times T1 and T2. The results obtained were minimum concentration (C _min_ ) for Group ED (n=12) median of 21.30mg/L (1.7-271.97 for minimum and maximum values) and coefficient of variation (CV) of 75%, whereas for Group DA (n=9), the values were 12.83mg/L (3.25-28.57) and 14.6%, (p=0.650, Mann-Whitney test), respectively.

Although there was no statistical difference between the groups, it is important to highlight the Group ED presented greater variability in both inefficacy and toxicity limits ^( 5 )^ as compared to Group DA. Therefore, 42% (n=5) of patients from Group ED with C _min_ over ten-fold the MIC of 8mg/L versus 22.2% (n=2) in Group DA. Furthermore, C _min_ was greater than the toxic concentration of 150mg/L in 33.3% (n=4) of patients in Group ED versus 0% in Group DA. Simultaneously, in Group ED, 25% (n=3) of patients were below 50%fT>MIC, as compared to 11% (n=1) in Group DA.

In addition, to use dose adjustment software in an efficient manner, some practical limitations observed when carrying out the study should be addressed and discussed, besides those previously presented by Silva et al., ^( 1 )^ The limitations are difficulty in estimating weight and height in critical patients at intensive care units (ICU), and in these cases, formulas not validated for critical patients are used; lack of confidence to work with DA, for both nursing team, ^( 1 )^ and prescribers’ team, who routinely use standardized antimicrobial doses recommended by manufacturers; and increased direct costs of medications, due to no intravenous mixture unit available for aseptic dilution, considering the fractionated vials were discarded, which did not contribute to reducing costs in the unit.

It is worth mentioning that these limitations could be extrapolated to a wide variety of services available in the country, and it is crucial that structural adjustments and appropriate work processes be implemented to expand the use of such technologies. Finally, the use of the software in the studied population provided less variability in plasma concentrations and in PK/PD correlation, suggesting clinical safety in its employment. However, further studies are required, with larger samples and direct and indirect cost analyses of software implementation, taking into account the short-, medium- and long-term scenarios, and the availability of an intravenous mixture unit for aseptic dilution, to avoid unnecessary losses.
