# Supplementary material for: Software for dose adjustment of antimicrobials. Implications for plasma concentrations and pratical limitations
Source: Einstein (Sao Paulo). 2020 Oct 20;18:eCE5858. doi: 10.31744/einstein_journal/2020CE5858 (PMC7575038; doi:10.31744/einstein_journal/2020CE5858)
Supplement: Translation

# Translation: Software para ajuste de doses de antimicrobianos. Implicações nas concentrações plasmáticas e limitações práticas

**Authors:** Geisa Cristina da Silva Alves, Samuel Dutra da Silva, Farah Maria Drumond Chequer, Whocely Victor de Castro, André Oliveira Baldoni, Andras Farkas, Gergely Daróczi, Jason Alexander Roberts, Cristina Sanches

**Affiliations:** 1 Brasil Universidade Federal de São João del-Rei, Divinópolis, MG, Brasil .; 2 Brasil Universidade de Itaúna, Itaúna, MG, Brasil .; 3 Estados Unidos Optimum Dosing Strategies, Bloomingdale, NJ, Estados Unidos .; 4 Austrália University of Queensland, Queensland, QLD, Austrália .

Caro Editor,

Esta carta visa complementar os dados publicados por Silva et al., ^( 1 )^ apresentando os resultados das concentrações plasmáticas de pacientes que utilizaram piperacilina (PIP) em dose empírica (DE) ou dose ajustada (DA) pelo software *Optimum Dosing Strategies* (ID-ODS), além de expor alguns pontos limitadores observados pelos autores durante a utilização do software na realidade de um hospital brasileiro.

Atualmente, existem disponíveis mundialmente alguns softwares para ajuste de dose de antibióticos, que utilizam dados específicos do paciente em conjunto com modelos populacionais farmacocinéticos. ^( 2 , 3 )^ Dessa forma, na ausência de dados individuais de concentrações plasmáticas, estes softwares podem estimar doses individualizadas com alta capacidade e acurácia para atingir concentrações-alvo. ^( 2 , 3 )^ Além disso, podem ser facilmente integrados ao prontuário médico para facilitar o manejo clínico do paciente crítico à beira do leito. ^( 2 , 4 )^ Assim, Silva et al., ^( 1 )^ realizaram a tradução e a validação linguística e cultural de um software de ajuste de dose, com posterior ensaio clínico randomizado comparando o desfecho entre os Grupos DE e DA para a PIP. O software foi configurado para o alvo farmacocinético/farmacodinâmico (PK/PD), com 50% do tempo em que as concentrações livres ficaram acima da concentração inibitória mínima (50%fT>CIM) e uma CIM de 8mg/L.

Os autores não observaram diferenças nos desfechos clínicos entre os grupos. Durante o estudo, foram coletadas no quinto dia de tratamento de PIP três amostras de sangue no mesmo intervalo de dose, as quais foram posteriormente processadas e analisadas. Para avaliar-se a eficácia microbiológica, a %fT>CIM foi calculada considerando a ligação de proteínas plasmáticas de 30% e extrapolando a curva de decaimento plasmática até a CIM de 8mg/L, a partir da fórmula K _el_ = (lnC _1_ -lnC _2_ )/(T _2_ -T _1_ ), sendo Kel a constante de eliminação e C1 e C2 as concentrações plasmáticas nos tempos T1 e T2. Como resultado, obtiveram-se concentração mínima (C _min_ ) para o Grupo DE (n=12) mediana de 21,30mg/L (1,7-271,97 para valores mínimo e máximo) e coeficiente de variação (CV) de 75%, enquanto, para o Grupo DA (n=9), os valores foram 12,83mg/L (3,25-28,57) e 14,6%, (p=0,650, Mann-Whitney), respectivamente.

Embora constatada ausência de diferença estatística entre os grupos, é importante destacar que o Grupo DE apresentou maior variabilidade nos dois extremos de ineficácia e toxicidade ^( 5 )^ em relação ao Grupo DA. Dessa forma, observaram-se 42% (n=5) dos pacientes do Grupo DE com C _min_ superior a dez vezes a CIM de 8mg/L versus 22,2% (n=2) no Grupo DA. Além disso, a C _min_ se encontrava acima da concentração tóxica de 150mg/L em 33,3% (n=4) dos pacientes do Grupo DE versus 0% do Grupo DA. Concomitantemente, no Grupo DE, 25% (n=3) dos pacientes estavam abaixo de 50%fT>CIM, em comparação com 11% (n=1) em DA.

Adicionalmente, para que a utilização de softwares de ajuste de dose seja eficiente, algumas limitações práticas observadas na execução do estudo precisam ser abordadas e discutidas, além daquelas previamente apresentadas por da Silva et al., ^( 1 )^ São elas: dificuldade de se estimar o peso e a altura em pacientes críticos em unidades de tratamento intensivo (UTIs) sendo, nestes casos, utilizadas fórmulas não validadas para pacientes críticos; insegurança em trabalhar com DA, tanto para a equipe de enfermagem, ^( 1 )^ quanto para a equipe de prescritores, que habitualmente utilizam doses de antimicrobianos padronizadas e preconizadas pelo fabricante e aumento dos custos diretos com medicamentos, devido à inexistência de central de misturas intravenosas para diluição asséptica, visto que os frascos fracionados eram descartados, o que não contribuiu para redução de custos na unidade.

Cabe ressaltar que essas limitações podem ser extrapoladas para uma ampla realidade de serviços existentes no país, sendo imprescindível que ajustes estruturais e adequação dos processos de trabalho sejam sanados para que se amplie o uso dessas tecnologias. Finalmente, a utilização do software na população estudada propiciou menor variabilidade nas concentrações plasmáticas e na correlação PK/PD, sugerindo segurança clínica em sua utilização, mas são necessários novos estudos, com maior tamanho amostral e com análise de custos diretos e indiretos da implementação do software, considerando o cenário de curto, médio e longo prazo e, ainda, a existência de uma central de misturas intravenosas para diluição asséptica, para se evitarem perdas desnecessárias.
